# Infection burdens and virulence under heat stress: ecological and evolutionary considerations

**DOI:** 10.1098/rstb.2022.0018

**Published:** 2023-03-27

**Authors:** T. E. Hector, A.-L. M. Gehman, K. C. King

**Affiliations:** ^1^ Department of Biology, University of Oxford, Oxford, Oxfordshire OX1 3SZ, UK; ^2^ Hakai Institute, End of Kwakshua Channel, Calvert Island, BC Canada, V0N 1M0; ^3^ Institute for the Oceans and Fisheries, University of British Columbia, 2202 Main Mall, Vancouver, BC Canada, V6T 1Z4

**Keywords:** thermal tolerance, pathogen evolution, virulence, transmission, trade-off, disease burden

## Abstract

As a result of global change, hosts and parasites (including pathogens) are experiencing shifts in their thermal environment. Despite the importance of heat stress tolerance for host population persistence, infection by parasites can impair a host's ability to cope with heat. Host–parasite eco-evolutionary dynamics will be affected if infection reduces host performance during heating. Theory predicts that within-host parasite burden (replication rate or number of infecting parasites per host), a key component of parasite fitness, should correlate positively with virulence—the harm caused to hosts during infection. Surprisingly, however, the relationship between within-host parasite burden and virulence during heating is often weak. Here, we describe the current evidence for the link between within-host parasite burden and host heat stress tolerance. We consider the biology of host–parasite systems that may explain the weak or absent link between these two important host and parasite traits during hot conditions. The processes that mediate the relationship between parasite burden and host fitness will be fundamental in ecological and evolutionary responses of host and parasites in a warming world.

This article is part of the theme issue ‘Infectious disease ecology and evolution in a changing world’.

## 1. Introduction

Global climate change has triggered escalating thermal variability and increased periods of extreme warm temperatures [1]. How individuals and populations, particularly ectothermic organisms, respond to the magnitude and pace of thermal variation is key to their persistence [2–4]. Gradual changes in average temperatures are slow relative to most biological processes [5]. By contrast, thermal variability over shorter time scales, such as seasonal, diurnal or tidal changes, are relatively fast [2,6]. Rapid weather-driven warming—heatwaves—can arise over a matter of days or even hours [4,5,7,8]. Extreme heat may disproportionately interfere with population and community structure and constitute the greatest selective force on species under continuing global climate change [4,9,10].

Alongside shifts in temperature, the geographical distribution of many parasites (including microbial pathogens such as viruses, bacteria and fungi) and the severity of infection during disease outbreaks are changing (e.g. [11,12]). Many populations are therefore facing the simultaneous stresses of extreme heat and virulent infection [13,14]. Although these environmental conditions may influence all host–parasite interactions in some way [15], much of the work to date focuses on ectothermic hosts and their parasites (table 1). The fact that infection can significantly increase the sensitivity of hosts to heat stress amplifies the risk that many populations face (reviewed in [14,26]). Indeed, infection can alter a host's entire thermal performance curve, shifting lower and upper thermal limits, alongside thermal optima (e.g. [27–29]). Exposure to parasites in combination with rapid heating may propel many species to the brink of extinction [30].

**Table 1.** Studies measuring the relationship between parasite burden and changes in host tolerance to extreme heat. Most studies find no relationship between host heat tolerance and parasite burdens (highlighted in bold). Studies were included if they quantified critical thermal maximum (CT_max_) or survival times following heat shock alongside quantifying parasite burden in infected individuals. To find these studies, a non-formal search of the literature was conducted (using Google Scholar search terms: (infect* OR parasite* OR pathogen*) AND (burden OR load) AND Ctmax), and forward and backward searches on known papers). While the list may not be exhaustive, it is likely representative. Only 10 studies were found highlighting the general lack of data available.

host type	host species	parasite type	parasite species	heat tolerance trait measured	type and duration of heat stress.	impact of infection on host heat tolerance	relationship between infection burden and host heat tolerance	notes on response	reference
honeybee	hybrid from *A. m. ligustica* and *A. m. carnica*	mite	*Varroa destructor*	TDT (thermal death time)	acclimation at 32°C or 38°C; static heat shock at 45, 47, 49 and 51°C	reduced TDT (38°C acclimation) or no difference (32°C acclimation)	**no clear difference in TDT of bees infected with 1 or 2 *Varroa* mites**	reduction only became apparent at warmer temperatures, in some cases a single mite infection increased TDT	Aldea-Sánchez *et al.* [16]
brown trout	*Salmo trutta*	myxozoan endoparasite	*Tetracapsuloides bryosalmonae*	CT_max_ (loss of righting)	0.22°C/min temperature ramp	reduced CT_max_	weak negative relationship	stronger negative relationship was found between CT_max_ and a symptom of infection (kidney hyperplasia)	Bruneaux *et al.* [17]
amphibian	*Litoria spenceri*	fungus	*Batrachochytrium dendrobatidis*	CT_max_ (loss of righting, or onset of spasms)	1°C/min temperature ramp	reduced CT_max_	**no relationship**		Greenspan *et al.* [18]
freshwater crustacean	*Daphnia magna*	bacteria	*Pasteuria ramosa*	CT_max_ (mortality)	0.06°C/min temperature ramp	reduced CT_max_	**weak negative & no relationship depending on genotype**	the strength of the relationship was dependent on host and pathogen genotype combination.	Hector *et al.* [19]
freshwater fishes	*Lepomis macrochirus* & *Lepomis megalotis*	helminth endoparasite	fish were parasatized with up to seven different helminth species	CT_max_ (onset of spasms)	1°C/min temperature ramp	no uninfected control to compare to	negative correlation	wild-caught fishes all with natural infections	Lutterschmidt *et al.* [20]
aphid	*Acyrthosiphon pisum*	fungus	*Beauveria bassiana*	CT_max_ (locomotion stopped)	0.3°C/min temperature ramp	reduced CT_max_	**no relationship**	there was no difference in the magnitude of changes in heat tolerance of hosts inoculated with a low or high parasite dose	Porras *et al.* [21]
beetle	*Hippodamia convergens*	fungus	*Beauveria bassiana*	CT_max_ (locomotion stopped)	0.3°C/min temperature ramp	reduced CT_max_	**no relationship**		
amphibian	*Notophthalmus viridescens*	protist	Ichthyophonus	CT_max_ (onset of spasms)	1°C/min temperature ramp	reduced CT_max_	**no relationship**	lesions on skin were measured as an index of infection load	Sherman [22]
mosquito	*Aedes aegypti*	virus	dengue virus	knockdown time	static heat shock at 42°C	reduced host knockdown times	**no relationship**		Ware-Gilmore *et al*. [23]
amphipod crustacean	*Corophium volutator*	trematode parasite	metacercaria	LT50: temperature causing 50% mortality	static heat shock for 10 min at 36, 36.5 or 38.5°C	marginal reduction in LT50	**no relationship**	infection burdens varied between approximately 1–20 parasites	Meißner *et al.* [24]
freshwater crustacean	*Daphnia magna*	bacteria	*Pasteuria ramosa*	knockdown time	static heat shock at 37°C	reduced host knockdown times	positive, negative, and **no relationship** depending on genotype	direction and strength of relationship depended on the host–parasite genotype combination, but there was no interaction with host sex	Laidlaw *et al.* [25]

**Figure 1.**
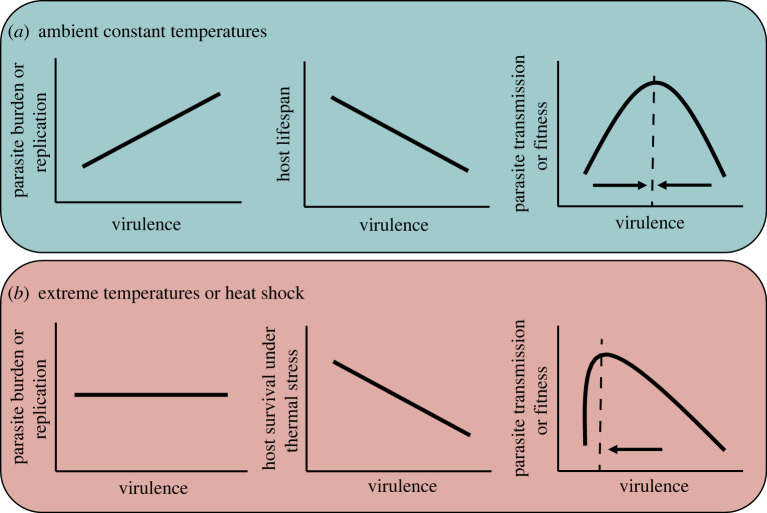
Virulence–transmission trade-off under ambient and stressful conditions. (*a*) Theoretical predictions from the trade-off hypothesis for the relationships between virulence and within-host parasite burden, host lifespan and parasite fitness—*R*_0_ (box 1). (*b*) Under extreme heat, the relationship between virulence and within-host parasite burden disappears, which has the potential to shift the relationship between virulence and parasite transmission. Without a positive relationship between parasite burden/replication and virulence, one consequence could be for lower virulence to become optimal (particularly when demographic change is considered [54]). However, if virulence is not associated with within-host parasite burden (or at least fitness), parasite evolution could instead be constrained or dampened due to the random removal of genetic variation within the population. Note that these hypothetical predictions do not account for important complexities of host–parasite systems, such as transmission mode, which mediate the relationships between host and parasite fitness traits ([40,42,44,45]; see main text and box 1 for a discussion of the generality of the trade-off hypothesis).

Parasites can significantly contract host heat tolerance during extreme warming events (e.g. [18,19,21,23]). Reduced tolerance to heating is therefore an intrinsic component of the virulence—harm caused by infection (e.g. host mortality and reduced reproductive output)—experienced by a host. Conventional wisdom predicts that parasites should experience a trade-off between virulence and transmission, driven in part by a positive relationship between virulence and parasite replication ([31,32]; box 1; figure 1*a*). However, within-host parasite burden, a common proxy for parasite replication and fitness, is rarely found to correlate with the harm hosts experience during heat stress (table 1). The general lack of a relationship between parasite burden and host heat tolerance is therefore puzzling. It suggests that host heat tolerance is influenced by interactions between several host and parasite processes. Heatwaves and rapid heating may therefore constitute a condition that negates the assumed link between parasite replication and virulence (e.g. [55]; box 1)*.* Disentangling the link between host and parasite performance in the face of extreme warming is crucial as these processes will fundamentally shape the eco-evolutionary responses of parasites to global change [13,14].

Box 1. **Virulence–transmission trade-off and parasite evolution**

Virulence can be defined as the harm parasites cause to a host during infection (reviewed by [33]). Virulence is highly variable across parasite species [33,34], with some species causing little harm to their host (e.g. *Henipavirus* infections in grey-headed fruit bats [35]) and some death (e.g. *Batrachochytrium* dendrobatids in amphibians [36,37]). Parasites are often dependent on their host for survival, but to be successful, parasites must transmit between new hosts [38]. For parasites, a trade-off is predicted to occur between within-host replication and between-host transmission [39–42]. To replicate, parasites consume host resources, which increases the harm caused to the host, reduces the host's lifespan and increases the risk of host mortality. The increased host mortality associated with replication is hypothesized to decrease parasite transmission [39–43], resulting in conflict between within-host growth and between-host transmission.

Overall, theory predicts that optimal virulence is that which maximizes transmission [33,39]. The virulence–transmission trade-off may be influenced by transmission mode [44]. For environmentally transmitted parasites, where transmission may be airborne or use vectors (indirectly transmitted [44]), high virulence is predicted to evolve as parasites do not rely on hosts for transmission [40,42,44,45]. Alternatively, parasites transmitted through direct contact between hosts (direct transmission [44]) rely on a live host for transmission. Thus, as these parasites rely on their host to successfully transmit to new hosts, theory predicts that extreme virulence is less likely to evolve [44,46].

Although empirical studies across a range of species find support for the virulence–transmission trade-off (e.g. [47–50]), other studies find contradicting evidence [31,50,51]. For example, if most of the virulence stems from toxin production and not pathogen replication, and there is a multiplicity of infection in the host, a clear relationship between growth and virulence would not be observed [32,52]. Infection by opportunistic parasites may also not yield this relationship as the interaction is not under natural selection [53]. Thus, the broad relevance and application of the virulence–transmission trade-off have been extensively debated and challenged [31,32,51]. A recent meta-analysis across empirical studies found strong support for a positive correlation between within-host replication and virulence and between within-host replication and transmission [43]. The authors also highlighted the need for further studies to be conducted to more accurately assess the relationship [43].

Here, we aim to understand the disconnect between within-host parasite burden and host performance during heating events. We will describe how the characteristics of host–parasite interactions, which vary widely across systems, may regulate the relationship between within-host infection burden and host heat tolerance. We propose a variety of mechanisms that could be operating when an infected host experiences excessive heat. It is a matter of urgency that we understand how changing global temperature patterns will influence the ecology and evolution of infectious diseases [56]. By bringing together knowledge from disease ecology and evolution, we hope to encourage the formation of predictions under extreme warming.

## 2. Host thermal performance as a component of parasite virulence

Evolutionary theory predicts a strong association between parasite virulence (usually modelled as parasite-induced mortality) and transmission (figure 1*a*; for virulence–transmission trade-off primer and a discussion of its general relevance see box 1). Optimal virulence is predicted to result from a trade-off between within-host replication and transmission duration (box 1; figure 1*a*) [33,39]. If we consider the reduction in host heat tolerance (e.g. increased host mortality during heat stress) as a component of parasite virulence, we may therefore predict a negative correlation with within-host infection burden (or replication rate). Despite this prediction, the relationship between host heat tolerance and infection burden, across a diversity of systems, appears to be weak—if not absent (table 1). Under extreme warming, therefore, the optimal strategy for a parasite may shift (figure 1*b*; [54]). It is potentially challenging to make predictions on parasite virulence evolution during heating based on the trade-off hypothesis (see box 1 for some caveats relating to the trade-off hypothesis).

Translating virulence into component traits can be tricky. One difficulty arises because virulence, as formalized in theory, is formidable to accurately measure as it relies on another abstract trait: fitness [31]. It is also often unclear which host–parasite traits interact to determine experienced virulence ([31]; box 1). Whether we consider virulence as a population or individual trait can also add confusion, particularly when traversing the fields of ecology and evolution. Parasites impact individual hosts, but evolution is a population-level process. So, it is necessary to translate processes which occur at the level of the individual into population-level responses [57].

## 3. The disconnect between host heat tolerance and infection burden

The extent to which infection can modify host performance during heat stress is vast. Infection can reduce host upper thermal limits, in some systems up to 8°C, and on average 2–3°C (Reviewed in [26]; see references in table 1). Within-host parasite burden, however, has rarely been found to correlate with the reduction in host heat tolerance caused by infection (table 1; box 2). In the mosquito *Aedes aegypti*, no correlation was found between reduced host heat tolerance and dengue virus burden, despite within-host variation in viral loads spanning two orders of magnitude [23]. The lack of a clear link between parasite burden and reductions in host heat tolerance demonstrates that other components of host–parasite interactions are key in regulating this aspect of virulence (see also [14,26]).

Box 2. **Obligate killers allow us to accurately link within-host parasite burden, parasite replication, virulence and transmission**

For obligately killing parasites, host mortality is an essential component of their transmission [58]. Selection on these parasites should maintain virulence that promotes host death at a time that maximizes transmission (see box 1; [55,59,60]), although trade-offs and classic assumptions may still apply [61]. For our interests, obligate killers are interesting because all parasite propagules remain within the host until after death. The relationships between parasite replication, parasite burden, virulence and transmission are much easier to characterize compared to non-obligately killing parasites. While obligate parasites may continue to replicate within a dead host, such replication would be significantly resource limited, and of minor concern in experimental studies in which hosts are frozen shortly after death. Obligately killing parasites thus present a powerful system for exploring the factors driving the relationship between within-host parasite replication and host heat tolerance.

*Pasteuria ramosa* is an obligate bacterial parasite of the freshwater crustacean, *Daphnia magna* [62]. *Pasteuria* spores exist in the environment and are picked up by the host during filter feeding. After entering a host, *P. ramosa* replicates, manipulates host physiology (causing castration and gigantism), reduces host fecundity and eventually causes early host death [63,64]. At the point of host death, millions of parasite transmission spores are released into the environment where they can persist until they reach the next host [60,63,64].

**Figure 2.**
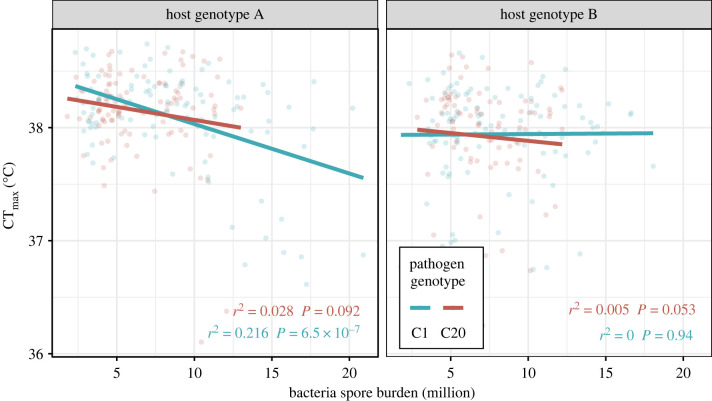
The impact of within-host *Pasteuria ramosa* spore burdens on the critical thermal limit (CT_max_) of *Daphnia magna* from Hector *et al*. [19]. CT_max_ is the temperature causing mortality during a 0.06°C/min heating ramp from ambient temperature and was measured on two host genotypes (A or B) infected with one of two parasite genotypes (C1 or C20). The strength of the relationship depended on the specific host–parasite genotype combination—a clear negative relationship is only apparent for one genotype pair.

*Pasteuria ramosa* imposes high levels of virulence under warming via substantial reductions in host heat tolerance [19,25]. Once infection becomes fully developed hosts can experience a reduction in their heat tolerance of up to 2°C. Within-host infection burdens spanning an order of magnitude (2–20 million) explained only a portion of this reduction (figure 2). A clear negative relationship was found in one host–parasite genotype combination linking these two traits (host genotype A + parasite C1; figure 2). Variation across host and parasite genotypes (figure 2) reveals how parasite burden (and replication) alone cannot fully explain reduced host heat tolerance during infection.

Host heat tolerance was also found associated with host body size and the age of an infection in this host–parasite system [19]. Host body size and infection age both represent highly quantitative traits. Interactions between host traits, in addition to genotype–genotype interactions, confirm that both the host and parasite are fundamental in driving host responses to heat stress.

Host–parasite systems are fascinatingly diverse [58]. This diversity, however, can restrict our ability to make broad generalizations. The language used to describe infection burdens varies considerably as a result (e.g. disease burden, spore loads, viral titre, infection intensity, etc.). For our purposes, we define burden as the number of parasite individuals infecting a host (although this definition itself has ‘grey areas’, e.g. box 3). Often these language differences indicate differences in host–parasite life histories and transmission strategies that will influence the relationship between host and parasite fitness traits.

Box 3. **When parasite replication and within-host burden are decoupled**

**Figure 3.**
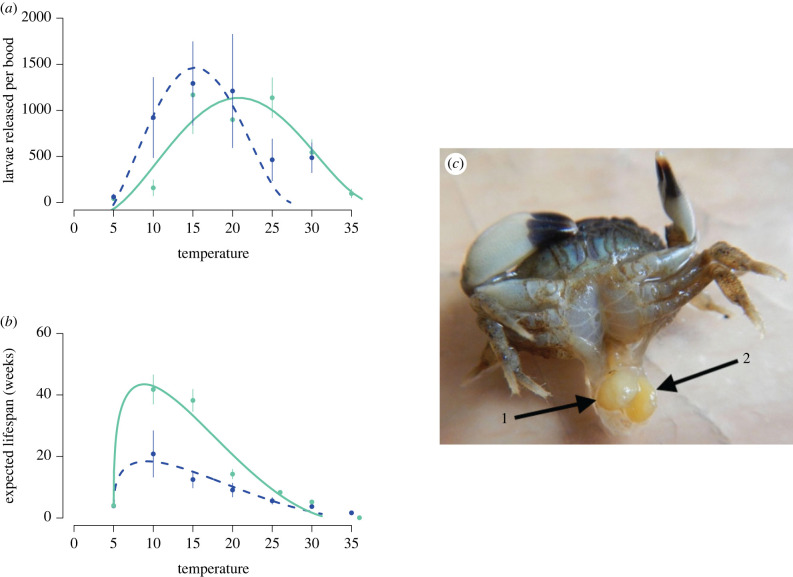
(*a*) The mean number of parasite larvae (i.e. nauplii and cyprids) released by brood from *Eurypanopeus depressus* infected by *Loxothylacus panopaei* with either one (solid line, estimate +/– s.e. of minimum temperature = 6.01 + /– 2.3, and maximum temperature = 38.03 + /– 2.34) or two externa (dashed line, estimate + /–s.e. of minimum temperature = 5.14 + /– 1.23 and maximum = 27.47 + /– 0.07) within a two-week period, after having been acclimated to temperature treatments over 11 days and held at experimental temperature for a week (mean + /– s.e.). Two-week period for comparison was selected because at 20°C, *L. panopaei* will release approximately 1 brood a week. (*b*) The expected lifespan in weeks of *E.* *depressus* infected by *L. panopaei* with either one (green, estimate + /– s.e. of minimum temperature 4.99, and maximum temperature 32.10 [27]) or two externa (dashed line, estimate + /–s.e. of minimum temperature = 4.99 + /– 0.02 and maximum temperature = 35.05 + /– 2.95). Expected lifespan was calculated from a fit survival object, methods available in [27]. Due to logistical constraints, replication in the two externa groups was not equal across temperatures (replication at 5°C = 3, 10°C = 5, 15°C = 4, 20°C = 3, 25°C = 3, 30°C = 4, 35°C = 3). Additional information about experimental design and methods in [27]. Animals with co-infections were kept in the same conditions as those with single infections. (*c*) An *E. depressus* with two attached *L. panopaei* externa (arrows indicate the two different externa). (Online version in colour.)

How parasite burden relates to changes in heat tolerance could depend on the extent of resource use by the parasite. In some metazoan parasites, reproduction and replication can be decoupled from parasite burden. For example, in long-lived metazoan parasites, the adult parasite may stay within a host for many years, producing many broods of parasite larvae (e.g. [65,66]). In this case, the parasite burden might be measured as the number of adult parasites within the host, and parasite replication the number of larvae the host is able to produce over a lifetime of infection. One example comes from rhizocephalan parasites that infect crab hosts [65]. While most infections are by a single parasite, in some systems there can be multiple infections by the same species within a host [67–69]. Co-infections can be visually detected by the existence of multiple reproductive organs, called externa (figure 3). It should be noted that not all multiple externa in rhizocephalans come from multiple individuals. In some species, a single individual can produce multiple externa in a single host [65].

Whether co-infection by multiple parasite genotypes leads to increased reproductive output, and how co-infection influences the thermal performance of the host and its parasites, remain open questions. The mud crab *Eurypanopeus depressus,* infected by the rhizocephalan *Loxothylacus panopaei*, can have co-infections [67,69], and single infection can lower host heat tolerance [27]. In addition to the experiments described in [27], parallel unpublished experiments with hosts with two externa were run (figure 3). The methods for hosts with multiple externa are the same as those for single externa [27]. Co-infections were naturally occurring in the field. Intriguingly, it appears that an increase in infection burden from one to two externas lowers the thermal performance optima and upper limit for parasite reproduction (figure 3). Infection by two externa also decreased the absolute rate of survival, but did not change the thermal dependence of survival (e.g. the shape of the survival response across temperature, figure 3). Despite that change in thermal performance, the overall total number of larvae produced by crabs infected with two externa was similar to hosts with single externa. Thus, co-infections led to a decrease in the per externa number of larvae. These results suggest there is a host-derived limit to the number of parasite larvae that can be produced. While we didn't see any increase in the relationship between temperature and survival between the one and two externa, the marked decline in expected survival suggests that the increase in incidence of infection has a direct cost. There is little benefit, and potentially a cost, to survival from co-infections, which could explain their relative rarity in the field [27].

For many unicellular parasites, such as bacteria and viruses, replication produces ‘offspring’ that can impact their host in a similar way to the ‘parent’ (e.g. by extracting resources, developing, replicating or interacting with the host's biology) [58]. In this group of parasites, within-host parasite burden could be reasonably proportional to virulence and transmission when parasite populations are large—particularly for parasites that transmit after host death (box 2; [60,70–73]). By contrast, some parasites transmit continually or in pulses while the host is alive (including both unicellular and multicellular parasites). If a host experiences lasting symptoms of infection from parasites after transmission, the negative impact on host heat tolerance may not be directly proportional to the parasite burden at the point of death (e.g. [17]). Similarly, for some multicellular parasites, replication and the parasite burden experienced by the host can be decoupled ([27], box 3).

Host responses to infection and heat stress may also play a vital role in mediating the relationship between parasite burden and thermal performance. Host heat tolerance might be governed by physiological or genetic trade-offs between responses to heat stress and to infection [74]. Across the sexes, genotypes and populations, hosts that exhibit the highest innate heat tolerance can suffer the greatest declines once infected (reviewed in [26]). In *Drosophila melanogaster*, immune activation alone was sufficient to reduce host heating tolerance, and this effect was most pronounced in heat-tolerant populations [75]. Infection has also been found to diminish sexual dimorphism by reducing heat tolerance of the more heat-tolerant sex [25]. Underlying genetic trade-offs for hosts could therefore mask any direct relationship between parasite burden and host tolerance to extreme heat [50].

Genetic variation in disease tolerance could also help explain the lack of a relationship between parasite burden and host heat tolerance. Rather than actively reducing parasite burdens (resistance strategy), a host may compensate for the fitness costs resulting from infection (i.e. disease tolerance; [76–81]). In the host *Daphnia magna,* the extent to which increases in *Pasteuria ramosa* parasite burdens reduced host heat tolerance varies across host genotypes. This pattern suggests genetic variation in host disease tolerance (box 2). The ability of a host to modulate its immune response between resistance and tolerance could also be thermally dependent. Indeed, thermal regulation itself has been suggested as a mechanism of disease tolerance [82]. From a parasite's perspective, virulence can be determined not only by total burden, but also by pathogenicity per parasite [83]. For some parasites, virulence is linked more closely to toxin production than replication [52], which may itself be mediated by parasite genetics and temperature [84]. Symptoms of infection (whether host- or parasite-induced) may become particularly potent under extreme heat [17]. Any relationship between infection burden and host heat tolerance could therefore be obscured if extreme heat promotes the expression of genetic variation for host disease tolerance, parasite pathogenicity or other drivers of virulence.

Infections progress through stages involving various host and parasite processes. Following initial infection by one or a few parasites, infections often develop via replication, use of host resources, host manipulation and extended periods of host defense [58,85]. The timing of heat stress relative to the stage of infection may determine which host or parasite infection processes are most influential to host heat tolerance. Some parasites may cause substantial costs to their host very early in infection by eliciting costly host defenses, causing immunopathology, or by some other physiological manipulation [75,86–89]. Opportunistic or recently emerged parasites, which may not rely on a specific host for transmission or may be maladapted, can in some cases impose high levels of virulence or illicit harmful immune responses [53,90]. In such cases, hosts could experience reduced heat tolerance simply as a result of initial infection, rather than any subsequent parasite replication.

Lastly, host heat tolerance may be less impacted by infection burden if temperature alters the colonization resistance that beneficial bacteria can provide to hosts. Microbes colonizing a host can confer protection against infection [91] and reduce parasite loads via direct or host-immune-mediated mechanisms (table 1 in [92]). An increase in the inhibitory effects of bacterial symbionts on parasites at higher temperatures has been shown in bumblebees [93] and mosquito vectors of malaria [94]. This pattern can extend to the protective effects of the host microbiota (community of host-colonizing microbial symbionts). Northern cricket frogs were found to be more resistant to infection by the invasive *B. dendrobatidis* at higher temperatures as these conditions promoted the persistence of the antifungal bacterium, *Stenotrophomonas maltophilia* on the skin microbiome [95]. The relationship between protection and temperature is not necessarily linear. In *D. melanogaster*, high temperatures resulted in greater *Drosophila* C virus replication and in lower *Wolbachia*-mediated protection [96]. However, in this system, temperatures during host development from egg to adult mediated bacteria-mediated protection. Higher temperatures (25°C) drove stronger protection, but protection was found to be lower or absent when flies developed at 18°C. Temperature-modulated manipulation of beneficial bacteria could be one explanation for a lack of a consistent relationship between parasite burden and host heat tolerance.

## 4. Host heat tolerance during infection: what mechanisms could be at work?

Across systems, host heat tolerance can be influenced by infection status (table 1). The underlying mechanisms driving these changes remain largely unexplored [14,21]. Given the wide range of host–parasite interactions that have similar responses, there could be multiple underlying mechanisms. Changes to host heat tolerance could occur as a result of the parasite's biological response (e.g. parasite resource extraction from the host), the host's biological response (e.g. immune response) or an interaction between the two species (e.g. tissue or mechanical damage to the host by parasite infection). To move from quantifying patterns to predicting future outcomes of heating events, we must understand which aspects of parasite infection can drive shifts in host thermal performance.

It is possible that changes in parasite resource extraction from its host could drive changes in host heat tolerance. The resources available to a parasite are directly linked to the pool of resources available to its host. How much host resource is given to the parasite can vary, depending on a range of factors, including the stage of infection, population density, host sex and manipulation of host physiology [97–102]. A host could increase its resource intake to accommodate the additional stress of infection (e.g. [103–105]). Interestingly, evidence from anthropogenic supplemental feeding on host–parasite interactions suggests that supplemental feeding can influence disease outcomes through individual and population-level changes in behaviour [106,107]. However, if an infection leads to a diseased state, then host energy intake could at times be reduced, potentially exacerbating the impact of infection burden on host performance (e.g. [108]).

The increased burden of providing energy for both parasite functions and its own can lead to an overall increased host energy use and metabolic rate (e.g. [99]). For example, a crab infected by a mature rhizocephalan can have double the metabolic rate of the uninfected host [109]. Similarly, in flies infected by ectothermic mites, there was an increase in metabolic rate that scaled with intensity of infestation [110]. Given a limit to a host's maximum metabolic rate [111], parasite-driven increases in host metabolic rate could cause hosts to reach their maximum rate at lower temperatures than uninfected hosts. The impact of infection on host metabolic rate will be a key determinant of host thermal performance. However, the relationship between within-host parasite burden and host metabolic rate may not always be linear [98,112,113], which could blur the link between parasite burden and host heat tolerance.

The strongest effect of infection on host performance can come around the thermal optima for parasite reproduction (e.g. [27]). Therefore, the increase in extraction of host resources used for parasite reproduction could be an underlying driver of reduced host performance. In the rhizocephalan-infected crab described above, the reduction in metabolic rate was only found in mature, reproductively active infections [109]. Additionally, in another species of rhizocephalan-infected crab, the strongest difference in the thermal performance between host and parasite was found at the optimal temperature for parasite reproduction, similar to that for uninfected host survival [27]. Where parasite reproductive output influences host thermal performance, infected host survival would be negatively related to the thermal dependence of parasite reproduction—which may not be proportional to within-host infection burden (see box 3). In systems where parasite burden is equivalent to parasite reproductive output, we may expect a stronger relationship between parasite burden and altered host–parasite heat tolerance (box 2).

Host immune response itself can be modulated by temperature and can have nonlinear relationships with temperature. For example, in an abalone and shrimp, there was a rapid decline in immune response at or above 32°C, making the host most susceptible to vibrio infections at high temperature [114,115]. In some marine invertebrates, immune response can increase with temperature [116], and yet the opposite is also true. Activation of the immune response itself can lead to changes in host heat tolerance [75]. In some cases of long-term macroparasite infections, there is evidence that some can inhibit or bypass the host's immune response [117]. Host heat tolerance could therefore be determined by potentially complex interactions between a host's own immune response and their current infection burden.

Beyond just the physiological tolerances of host and parasite, there are behaviours and physiological responses to infection that directly alter the experienced temperature for the system. In endotherms, fever can help to trigger immune responses [118] and generally have a strong influence on the host's thermal response. A fever response in endotherms, however, may limit their ability to regulate internal body temperatures during extreme heat events. In July 1995, 57% of patients admitted for near-fatal heat stroke were also carrying pathogenic infections [119,120]. In some ectotherms, behavioural changes, such as moving towards hotter micro-climates, can alter the host's body temperature to combat parasite infection, known as behavioural fever [121]. Interestingly, some species manipulate parasite reproduction through febrile behaviour by plastically regulating their temperature to below the parasite's optimum [122]. In longer-lived parasites that can manipulate host behaviour—for example, trematode worm infections in snail hosts—heat tolerance of infected hosts can be parasite species-dependent [123] and can lead hosts to select for thermal niches that may benefit the parasite [124].

Heating may also drive a disconnect between parasite burden and virulence by interacting with host and parasite acclimation and size differences. The capacity of organisms to increase their tolerance to heat stress via thermal acclimation is often size-dependent [74,125,126]. In addition, warmer temperatures drive increased rates of reproduction, but generally result in smaller offspring ([127–130]; see [13]). In some parasites, small cells can cause higher virulence as they more effectively evade immune responses [131], which may in part explain the observation that some parasites become more infectious following development at warmer temperatures [132]. The relationship between parasite burden and host heat tolerance could therefore be skewed across temperatures by size-dependent virulence, as well as the interaction between host and parasite acclimation capacity (e.g. [16,18]).

## 5. Parasite transmission strategy may direct the eco-evolutionary consequences of extreme heating

The transmission strategy of a parasite will shape its eco-evolutionary response to heating by regulating the relationship between parasite burden and virulence. Parasites can be broadly grouped by how they transmit between hosts. Important transmission strategies include parasites that transmit directly between alive hosts or via vectors, parasites that kill their host to transmit or have environmental transmission stages, and opportunistic parasites not dependent on a host [53,55,58,59]. To begin to understand the eco-evolutionary consequences of heating across the diversity of parasite strategies, it is useful to consider predictions formulated for other drivers of disease dynamics.

Both seasonality and predation share similarities with extreme heating, often involving rapid changes in host and parasite population sizes and asymmetrical mortality across healthy and uninfected hosts [133–140]. It seems likely, however, that extreme heating will impose a far more intense selective force than that of most seasonal changes and predation [4,9,10]. The pressures put upon populations by extreme heat and infection will be multifaceted, not only contracting population sizes due to excessive mortality [1,141–144] but also impacting subsequent population growth via reductions in reproduction and fertility [145–148]. The dynamics here may match closely to those seen in mosquito vector systems under extreme daily temperature fluctuations [149–152]. We highlight the need for the development of specific eco-evolutionary theory addressing the interaction between infection dynamics and parasite virulence under extreme heat [13,14,56].

**Figure 4.**
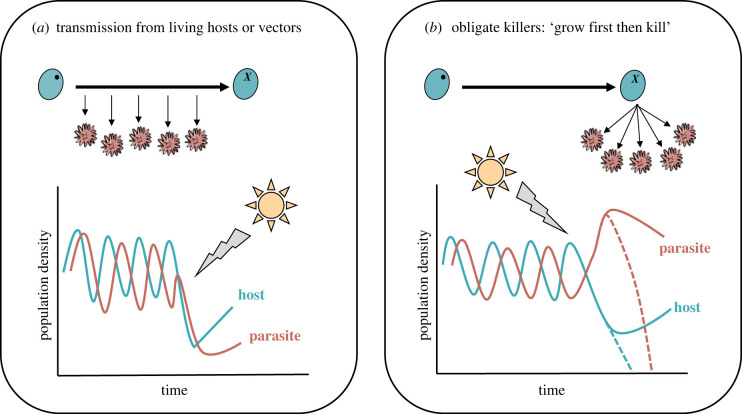
Ecological relationship between parasite transmission strategy and host–parasite population densities under extreme heating. (*a*) Direct transmission between living hosts or via vectors. (*b*) Transmission depends on host death or via environmental stages. Blue and red lines represent the dynamics of a hypothetical host and parasite population, respectively. Across time, we may see fluctuating host–parasite population dynamics depending on the system [153]—although our general point does not depend on the exact nature of these dynamics. After an extreme heat event (sun and arrow) population dynamics are interrupted, with subsequent dynamics depending on the host–parasite system: (*a*) excessive host mortality causes both host and parasite populations to shrink, with parasite reestablishment lagging behind any host population recovery; (*b*) excessive host mortality results in an overabundance of environmental parasite transmission stages, which could suppress host population growth or cause local extinction.

Ecologically, for parasites that transmit between living hosts (directly or via vectors), heating may tightly constrain their ongoing transmission (figure 4*a*). Disproportionate declines in the number of infected hosts due to heat stress, alongside the death of a portion of the susceptible host population (e.g. [141–144]), will shrink the susceptible and infected population sizes. As a result of extensive host mortality, the parasite population size (i.e. infected hosts) will also contract. Ongoing transmission following an extreme heat event could therefore be limited by both small host and parasite population sizes (figure 4*a*; e.g. [149,150,154]). Over time, host populations may slowly recover, with a lag in parasite transmission, potentially resulting in slow joint epidemiological dynamics as populations reestablish (figure 4*a*). However, if host populations (both infected and uninfected) are reduced too much they risk extinction, and transmission may fall below a threshold for parasite persistence [154].

In obligate killers and parasites that can persist in the environment extreme heat will cause high mortality in the infected host population (figure 4*b*). In contrast with other transmission strategies, however, the environment may become saturated with viable environmental parasite transmission stages. Based on theory from seasonal population disturbances, we expect that the subsequent ecological dynamics will depend on factors including the population sizes of both the surviving parasites and susceptible hosts, and the *R*_0_ of the parasite [38,133,155]. An overabundance of parasites in the environment, especially if they are highly virulent or transmit quickly, could either overwhelm the remaining host population causing local extinction, or suppress host populations preventing fast host population growth (figure 4*b*; [133,145,155–157]).

The evolutionary consequence for parasites with different transmission strategies may vary because of the distinct ecological dynamics discussed above (figure 4). In systems with direct or vectored transmission, both host and parasite populations may experience a bottleneck following extreme heating (figure 4*a*; [135]). Host and parasite evolution may simply be constrained by low genetic variation—without any shift in the parasite population's mean trait values. However, if heat extremes cause higher mortality for hosts infected with more virulent parasite genotypes (e.g. [19]), low virulence infections may continue to transmit, and selection could favour the evolution of reduced virulence (e.g. [137,–139,158]). Indeed, the demographic impact of heating may increase selection on virulence relative to transmission, driving the evolution of reduced virulence [54]. Importantly, if virulence experienced during heat stress is unrelated to within-host parasite burden, selection on virulence, replication and transmission could be decoupled [55].

When host populations infected with obligate killers face extreme heat, it is likely that ongoing transmission will be predominantly host limited [156]. The relatively large proportion of infected hosts that die will contract host population size and cause a large release of viable parasites into the environment [156]. Less virulent parasite genotypes whose hosts survive heat stress may have a fitness advantage as they continue to replicate. However, there will also be an accumulation of more virulent genotypes in the environment. Virulence evolution will therefore depend on the capacity of surviving hosts to reestablish their population [145,156] and the relative fitness advantage for parasites replicating in surviving hosts versus those already in the environment [55,60]. If virulence is unrelated to parasite burden selection on parasite fitness traits may become decoupled. The less virulent parasite genotypes whose hosts survive heat stress may not be constrained by a virulence–transmission trade-off (or selection on virulence may dominate, e.g. [54]). As a result, evolutionary dynamics for these parasites may not follow dynamics predicted from current theory (e.g. [60]).

## 6. Conclusion

While we are beginning to understand the consequences of infection for hosts during heating, the eco-evolutionary consequences for parasites remain unclear [13,14,27,56]. Trade-off theory predicts a strong relationship between within-host parasite burden and virulence (box 1). Under extreme heat, there is often only a weak relationship, if any (table 1). Considering the diversity of host–parasite systems, a broad definition of parasite burden is difficult, let alone establishing general explanations for the missing link between parasite burden and host heat tolerance. More empirical studies are needed, across a wider diversity of systems, to make broad predictions about host and parasites under heating. At a fundamental level, extreme heat may present a condition under which relationships assumed by general theory simply do not hold (box 1).

Ongoing transmission—except for obligate killers—during an infection may affect our capacity to measure the cumulative parasite burden that a host has experienced. Obligate killers present us with the opportunity to directly relate parasite burden to replication, virulence and transmission. In these systems, a relationship between parasite burden and host heat tolerance has been documented, showing that parasite replication is an important driver of host sensitivity to heat stress (box 2). It is clear, however, that other aspects of host–parasite interactions are also crucial. Interactions between host and parasite processes at the genetic, physiological and ecological levels will regulate the eco-evolutionary relationship between parasite burden and host performance [12–14,26,27,56]. Escalating heating events are going to be a driving force for the evolution of hosts and parasites.

There is a pressing need to extend our understanding about how heating will drive host–parasite eco-evolutionary dynamics [13,14,56]. The development of theory will help guide experimental efforts, allowing us to make more grounded and detailed predictions about the ramifications of heating for parasite evolution. To predict evolutionary responses to heating, the link between individual and population-level eco-evolutionary processes will need to be considered [50,57,60,140,159]. The relationships between parasite virulence and transmission under heating may be mediated by interactions between the genetics, physiology and transmission strategy of hosts and parasites [26]. How within-host interactions translate into population-level processes, such as host mortality rates, population dynamics and parasite fitness, will in turn drive evolutionary change to extreme heat [54,57,60,140,159]. Heating may also shift genetic trade-offs within and between parasite fitness traits, and the strength of selection on virulence and transmission, shaping parasite evolutionary potential [50,54,60]. Predicting parasite evolution will also be aided by a deeper understanding of the mechanisms underlying infection-related declines in host heat tolerance (e.g. CT_max_). Our discussion on mechanisms is predominantly speculative. Without understanding the mechanisms at play, it is difficult to know where our theoretical and experimental efforts should lie (e.g. [160]). Finally, to support modelling efforts, approaches such as experimental evolution of parasites across host populations in the laboratory could help to solidify evolutionary predictions.

Present predictions for disease transmission and distributional changes under global change do not incorporate the capacity for parasites to evolve (e.g. [12,27,161]). That under heating, key parasite infection traits do not align with conventionally predicted relationships (i.e. there is little relationship between parasite burden and virulence under heat stress), suggests current methods for prediction may be challenging to apply. A deeper understanding of these complex processes will be valuable for addressing the impacts of parasite evolution for host species persistence, host–parasite distributions and even the potential for zoonotic spill-overs into human and wildlife populations.

## Data Availability

Data presented in box 2 can be found at https://doi.org/10.1111/gcb.14713. Unpublished data presented in box 3 have been provided as electronic supplementary material. Data from Gehman *et al*. [27] can be found at https://github.com/alyssamina/Thermal-ecology-disease. The data are provided in electronic supplementary material [162].
